# Understanding general practitioners’ prescribing choices to patients with chronic low back pain: a discrete choice experiment

**DOI:** 10.1007/s11096-023-01649-y

**Published:** 2023-10-26

**Authors:** Melanie Hamilton, Chung-Wei Christine Lin, Sheena Arora, Mark Harrison, Marguerite Tracy, Brooke Nickel, Christina Abdel Shaheed, Danijela Gnjidic, Stephanie Mathieson

**Affiliations:** 1 Institute for Musculoskeletal Health, Level 10 North, King George V Building, Royal Prince Alfred Hospital (C39), Missenden Road, PO Box M179, Camperdown, Sydney, NSW 2050 Australia; 2 Sydney School of Public Health, Faculty of Medicine and Health, The University of Sydney, Sydney, Australia; 3 Centre for Health Economics Research and Evaluation, Faculty of Health, University of Technology Sydney, Sydney, NSW Australia; 4 Faculty of Pharmaceutical Sciences, University of British Columbia, Vancouver, Canada; 5 The Centre for Health Evaluation and Outcomes Sciences (CHEOS) at St. Paul’s Hospital, Vancouver, Canada; 6 Sydney School of Public Health, Faculty of Medicine and Health, The University of Sydney, Sydney, NSW Australia; 7 School of Public Health, Faculty of Medicine and Health, The University of Sydney, Sydney, Australia; 8 School of Pharmacy, Faculty of Medicine and Health, The University of Sydney, Sydney, NSW Australia

**Keywords:** Clinical decision making, Discrete choice experiment, Opioids, Primary care

## Abstract

**Background:**

Although NSAIDs are recommended as a first line analgesic treatment, opioids are very commonly prescribed to patients with low back pain (LBP) despite risks of harms.

**Aim:**

This study aimed to determine factors contributing to general practitioners’ (GPs’) prescribing choices to patients with chronic LBP in a primary care setting.

**Method:**

This discrete choice experiment (DCE) presented 210 GPs with hypothetical scenarios of a patient with chronic LBP. Participants chose their preferred treatment for each choice set, either the opioid, NSAID or neither. The scenarios varied by two patient attributes; non-specific LBP or LBP with referred leg pain (sciatica) and number of comorbidities. The three treatment attributes also varied, being: the type of opioid or NSAID, degree of pain reduction and number of adverse events. The significance of each attribute in influencing clinical decisions was the primary outcome and the degree to which GPs preferred the alternative based on the number of adverse events or the amount of pain reduction was the secondary outcome.

**Results:**

Overall, GPs preferred NSAIDs (45.2%, 95% CI 38.7–51.7%) over opioids (28.8%, 95% CI 23.0–34.7%), however there was no difference between the type of NSAID or opioid preferred. Additionally, the attributes of pain reduction and adverse events did not influence a GP’s choice between NSAIDs or opioids for patients with chronic LBP.

**Conclusion:**

GPs prefer prescribing NSAIDs over opioids for a patient with chronic low back pain regardless of patient factors of comorbidities or the presence of leg pain (i.e. sciatica).

**Supplementary Information:**

The online version contains supplementary material available at 10.1007/s11096-023-01649-y.

## Impact statements

- GPs preferring to prescribe an NSAID over an opioid for a patient presenting with chronic low back pain is consistent with recent shifts in opioid prescribing data, showing the increased prescribing of opioids is starting to decline for chronic pain conditions.
- Recent updates to clinical practice guidelines now discourage the prescribing of opioid analgesics to patients with low back pain. Findings from our study suggest greater general practitioner (GP) adherence to clinical practice guideline recommendations, and/or that GPs are more aware of the emerging evidence that opioids are no more effective than NSAIDs for low back pain.

## Introduction

Chronic (> 3 months) [1] low back pain (LBP) is a leading cause of disability globally [2], with significant impacts on society and individuals [3]. Patients with LBP commonly seek treatment from general practitioners (GPs, also known as family physicians) in primary care settings [4, 5]. Most cases of LBP are considered “non-specific,” as they are not attributed to a known cause, and 5–10% of people with LBP will also present with referred leg pain (sciatica) [6, 7].

International clinical practice guidelines [8–12] first recommend non-steroidal anti-inflammatory drugs (NSAIDs) for chronic LBP and recommend to only prescribe weak opioids for short periods in instances where NSAIDs or other treatments are contraindicated or ineffective [11–13]. However, despite increased awareness of the harms of opioids, such as dependence, overdose and death [14–17], data from Australia [18], the UK [19], the US and Canada [20] show a significant increase in the prescribing of opioid analgesics [21–27]. Additionally, there has been a decrease in the prescribing of NSAIDs and an increase in the prescribing of opioids to patients with chronic LBP in primary care [18], as well as in other chronic pain conditions [18–21]. This study is needed to help understand factors contributing to analgesics prescribing, particularly opioids, to inform strategies to reduce unnecessary overprescribing.

Qualitative studies reveal GPs’ choices for prescribing analgesics to patients with chronic pain may be influenced by factors, such as potential adverse events, the presence of multiple comorbidities, and consideration of the harms versus the benefits of improved pain relief [28–31]. Qualitative and observational methods identify the various factors which may generally influence choices and therefore contribute to the development of the attributes and levels used in a discrete choice experiment (DCEs). DCEs are a stated choice tool that quantitatively aims to understand reasons behind choices, with a particular focus on investigating factors which people are willing to ‘trade-off’ when making choices [32]. DCEs are particularly useful when there is no revealed preference data available, which is the case in there being a lack of data that describes GPs’ prescribing preferences for pain medications in the context of pain reduction and adverse events. Previous research in analgesic preferences [33, 34] using DCE methodology has found that patients suffering from chronic pain conditions are concerned of the potential harms associated with NSAIDs and opioids, particularly those related to cardiovascular, gastrointestinal and risks of dependency [35, 36]. Although, people are often still willing to accept some level of risk to achieve a significant reduction in pain [35, 36]. Such DCEs have not yet investigated clinician prescribing choices for patients with chronic LBP. Since GPs are central to prescribing analgesics medications for chronic LBP, understanding what influences the treatment choices of GPs is important, as such factors can have direct or indirect impacts on unnecessary prescribing and address concerns with the overuse of opioids [37].

### Aim

This study aimed to determine factors contributing to, and the degree to which they influence GPs’ prescribing choices to patients with chronic LBP in a primary care setting. Secondary outcomes included the willingness to accept risk of adverse events for effectiveness of medications and the preferences for analgesic options in various clinical scenarios.

### Ethics approval

Ethics approval (2020/738) was obtained from The University of Sydney Human Research Ethics Committee (HREC). Informed consent was obtained from participants via Qualtrics in the format of a signed electronic form.

## Method

### Study type/design

This study utilised a discrete choice experiment (DCE) methodology. The DCE technique is a quantitative attribute-driven measure of cost–benefit analysis, based on the assumption that decisions in health-care can be described by their attributes (e.g. the degree of pain relief provided by a medication) and that an individual's degree of preference depends upon the levels and their willingness to trade-off between attributes (e.g. considering the degree of pain reduction in combination with the potential number of adverse events). This DCE was delivered in an online survey format [38]. The survey provided a hypothetical representation of a real-life choice participants may be faced with and contained the minimum information needed for participants to distinguish between options. In a DCE, attributes and levels vary across the alternatives (i.e. the options within a choice set (question) with the overall goal being to determine which attributes are influencing clinical choices. This DCE considered three clinical attributes (drug type, degree of pain reduction and number of adverse events) and two patient attributes (LBP with or without referred leg pain (sciatica) and comorbidities). The attributes and levels are presented in Table 1. The reporting of this DCE follows the checklist of conjoint analysis applications in healthcare [39] as incorporating these values in GP decision making may assist in aligning health care policy with clinician preferences and improve the effectiveness of health care interventions.

**Table 1:** Clinical attributes and their levels for each alternative

Attribute	Alternatives		
	Levels for the opioid option	Levels for the NSAID option	Neither option (opt-out)
Drug			
	Oxycodone hydrochloride, 1 × 5 mg capsule every 6 h	Meloxicam 15 mg, once daily	No medication
	Oxycodone controlled release, 1 × 5 mg tablet (+ naloxone 2.5 mg) every 12 h	Diclofenac 50 mg, twice daily	
	Tramadol hydrochloride, 1 × 50 mg capsule, every 6 h		
	Codeine phosphate 30 mg + paracetamol 500 mg, 2 tablets every 4–6 h		
Degree of pain reduction			
	Pain intensity reduced to 5 out of 10	Pain intensity reduced to 5 out of 10	Pain intensity remains the same
	Pain intensity reduced to 4 out of 10	Pain intensity reduced to 3 out of 10	
Number of adverse events			
	7 in 10 patients experience mild adverse events	4 in 10 patients experience mild adverse events	No adverse events
	4 in 10 patients experience mild adverse events	2 in 10 patients experience mild adverse events	

The opioid and non-opioid options are presented randomly in alternate order between choice sets

### Study population and recruitment

GPs were recruited from an Australian panel of registered doctors on Qualtrics research services. Qualtrics is a survey management company that is experienced in recruitment and conducting surveys including those of DCE design (https://www.qualtrics.com/uk/what-is-qualtrics/). There were no exclusion criteria for the participants except GPs who had completed the pilot survey (further details provided in Sect. 2.5 below) were not invited to complete the final DCE survey.

### Development of the labelled DCE design

The development of the clinical scenarios, attributes and levels were based on previous research on back pain and medicine prescribing in general practice in Australia [18, 21, 28–31, 40–42]. Although paracetamol has been commonly prescribed for LBP in the past, recent evidence has found it to be no more effective than placebo and hence it was not chosen for inclusion [43, 44]. We included opioids and NSAIDs as the drug alternatives as they are the most commonly prescribed analgesics for LBP in Australia [18, 21]. The selection of the different types of opioid analgesics and NSAIDs within each drug class were based on our previous research of the most common medicines and dose prescribed for spinal pain in general practice in Australia [18], in addition to the literature on the different indications, modes of action, and dose recommendations which GPs prescribe for various pain intensities and conditions [18, 42, 45]. The clinical attributes of the degree of pain reduction and number of adverse events were selected based on evidence of recent systematic reviews of placebo-controlled trials of opioids and NSAIDs [46–48]. The evidence indicates the amount of pain relief provided by these two analgesic classes is similar [46–48]. The two patient attributes (LBP with or without referred leg pain (sciatica) and comorbidities) were selected based on discussions with clinical experts (GPs, pharmacists) and evidence from published literature [28–31, 41, 47, 49, 50] of what GPs believe to be important considerations associated with the management of chronic LBP. For instance, the management of patients with multi-morbidities is complex as it is often associated with polypharmacy and increased risks of harms [49, 50]. Additionally, GPs may be hesitant to prescribe NSAIDs to patients with gastrointestinal comorbidities [41, 47] and avoid prescribing opioids in instances where patients present with comorbidities, such as depression [28, 31]. The attributes and levels were agreed upon through discussion between investigators who are experts in pharmacy, primary care and health economics. The Johnson and Orme rule of thumb method [51] was used to guide the feasibility of the number of attributes and levels, which also provided the initial sample size estimate of 195 participants.

### Choice sets, attributes and levels

The DCE survey comprises a series of ‘choice set’ questions. The choice sets in this DCE were generated using a D-efficient, balanced design with N-Gene software [52]. The D-efficient design determined the order of the levels (all levels in patient and clinical attributes) within the choice sets and randomised the selection of choice sets allocated to each participant. The alternatives in each choice set were ‘unlabelled’ (i.e. labelled as option A or B, not labelled as opioid or NSAID). These aspects minimised potential bias by ensuring that each GP participant was presented with different combinations of the two patient attributes being; LBP with or without referred leg pain (sciatica) and number and type of comorbidities (gastric reflux, cardiovascular disease and/or depression). The ‘balanced design’ [53] aspect indicates that each attribute level occurred equally to minimise the variance in the parameter estimates and meant less participant responses were needed [54]. Table 2 provides an example of a choice set. A total of 60 choice sets were generated and these were divided into 5 blocks of 12, each participant was randomised to receive one of the 5 blocks. Participants received instructions prior to commencing the survey, explaining that they will receive a unique collection of twelve choice sets (i.e. questions). Each choice set will start with a clinical scenario that will represent a patient with low back pain of three months duration and self-reported pain intensity of 6 out of 10. Two patient related attributes will change in each choice set; low back pain presentation being with or without referred leg pain, and the presence of comorbidities (no comorbidities or no history of gastric reflux or current risk of cardiovascular disease or depression). Participants were required to choose their preferred alternative from three (i.e. what they would most likely to choose in clinical practice): an opioid analgesic, an NSAID, or an “opt out” (neither) alternative if participants felt neither the opioid nor the NSAID alternatives were preferable. The inclusion of the opt-out alternative allowed for participants to respond in a way that is consistent with how they would prescribe in a real-life situation, by not forcing them to choose and therefore, ensure greater external validity of the study results [55].

**Table 2:** Choice set question example

Jamie is a 49 year old patient who presents to your clinic with chronic non-specific low back pain. The low back pain started 3 months ago. The pain intensity is rated as 6 out of 10. There is no referred leg pain or neurological symptoms. There are no red flags (e.g. history of cancer, infection etc.) Jamie has tried paracetamol (4 g per day) and then ibuprofen (400 mg, four times a day) but with little relief. Jamie has stopped walking daily because of the pain but has been able to continue working. There is no history of gastric reflux or current risk of cardiovascular disease or depression
Jamie doesn’t smoke and drinks alcohol at safe levels. Body Mass Index (BMI) is 25. There is no history of substance abuse and has not been prescribed opioids in the past

Attribute	Option A	Option B	Opt-out
Medicine	Oxycodone hydrochloride One 5 mg capsule, taken every 6 h	Meloxicam One 15 mg tablet, taken once daily	
Pain reduction	Pain intensity reduced to 5 out of 10	Pain intensity reduced to 5 out of 10	
Adverse events	7 in 10 patients experience mild adverse events	4 in 10 patients experience mild adverse events	
My choice would be	□ Option A	□ Option B	□ Neither A or B

### Pilot study

A pilot test of our DCE design was conducted on the 24th of August 2021 and completed by 21 participants. The pilot study pretested the full survey to ensure the technical setup of the survey faced no issues and the choice set questions were correctly understood by the participants recruited from Qualtrics. At the end of the survey, each participant also completed some demographic questions (Supplementary material A1.1) and several process evaluation questions designed to obtain information about participants’ experience of completing the DCE tasks (Supplementary material A1.2) [56]. The questions and answers to the process evaluation confirmed no changes needed to be made to the survey before conducting the main study. The sample size calculation for DCEs is unique as it is based on the number of attributes and levels within the design. The sample size for our final design (n = 210) was higher than what was calculated for the pilot study as this was confirmed using the coefficients from the pilot study, based on standard DCE sample size calculation methods [51].

### Data collection

The DCE was delivered in the form of an online survey in Qualtrics. Participants, who were already registered as GPs on the Qualtrics platform completed the DCE between the 8th and 25th of November 2021. After completing the DCE, Participants also completed the following demographic questions; age, gender, country of education, years spent in general practice, number of GPs in their practice, if they have a special interest in managing musculoskeletal conditions, practice location, workload capacity and payment method for consultations. Qualtrics reimbursed GPs for their time.

### Analysis

Participant characteristics and responses to the process evaluation questions were summarised using descriptive statistics. Responses to the DCE were analysed using a mixed logit model in STATA [53]. The patient attributes (i.e. LBP with or without referred leg pain and comorbidities) were held fixed and considered as random variables, assumed to vary over a normal distribution. We analysed main effects only and generated coefficients, odds ratios and standard errors for all attributes and corresponding levels. An alternative specific constant (ASC) was included in the analysis to account for the opt-out alternative, which was the base alternative. The coefficient values indicate the significance of each attribute individually, while holding all other attributes in the model constant and were considered statistically significant at the 5% level, and tests of significance for the coefficients were two sided.

Through the analysis of the coefficients, we calculated the significance of each clinical attribute (i.e. the different drug types within each class of opioids or NSAIDS, the amount of reduction in pain intensity and the number of adverse events) and patient attribute (LBP presentation with or without referred leg pain and comorbidities) in influencing clinical decision making (primary outcome). We also investigated the degree to which GPs would prefer to choose an alternative either based on the number of adverse events or the amount of pain reduction (secondary outcome) by calculating direct and indirect marginal effects of pain and adverse events on each alternative. We also conducted a sensitivity analysis where we removed participants who only selected the “opt-out” alternative for every choice set and a post-hoc exploratory analysis of interaction effects of participant characteristics on medication choice.

## Results

A total of 1,607 clinicians accepted the invitation, 204 participants did not complete the survey, 141 did not consent and 1,052 were not eligible due to not being not GPs. As only eligible participants were able to complete the survey and it was a requirement to complete all questions, there was no missing data. Two hundred and ten remaining GPs completed the main study. Participants took on average 9 min to complete the survey. The mean age of respondents was 34.0 years (SD = 9.2), 67.6% were women, mean number of years practicing as a GP was 6.5 years, 47% had a special interest in managing musculoskeletal conditions and 59.5% worked full time. The demographic characteristics of the participants are presented in Table 3 and the feedback responses from the main study are reported in Supplementary material 2.

**Table 3:** Participant demographics

Participant characteristics	Number of general practitioners (%) (n = 210)
Age category	
< 35 years	136 (64.8%)
35–44 years	45 (21.4%)
45–54 years	20 (9.5%)
55 + years	9 (4.3%)
Gender	
Male	58 (27.6%)
Female	142 (67.6%)
Did not say	10 (4.8%)
Country of graduation	
Australia	180 (85.7%)
Other	25 (11.9%)
Did not say	5 (2.4%)
Years spent in general practice	
< 2 years	20 (9.5%)
2–5 years	107 (50.9%)
6–10 years	51 (24.3%)
11–19 years	18 (8.6%)
20 + years	14 (6.6%)
Number of general practitioners in the practice	
< 5	92 (43.8%)
> 5	118 (56.2%)
Location of practice*	
Major city	176 (83.8%)
Inner regional	13 (6.2%)
Outer regional	8 (3.8%
Remote	0 (0%)
Unknown	13 (6.2%)
Special interest in managing musculoskeletal conditions	
Yes	99 (47.1%)
No	111 (52.9%
Workload capacity (self-reported)	
Part-time	82 (39%)
Full-time	125 (59.5%)
Retired	3 (1.4%)
Payment for consultations**	
Majority Bulk billed	123 (58.6%)
Majority charged a gap payment	25 (11.9%)
Both	62 (29.5%)

*The location of practice was based on the Australian Statistical Geography Standard from The Australian Bureau of Statistics. Volume 5-Remoteness Structure, July 2016. https://www.abs.gov.au/ausstats/abs@.nsf/Latestproducts/1270.0.55.005Main%20Features20July%202016?opendocument&tabname=Summary&prodno=1270.0.55.005&issue=July%202016&num=&view=

**Australia has a universal public health system (Medicare) therefore, many patients will not pay out of pocket to see a GP (Bulk billing). However, some GPs do charge additional fees on top of the Medicare rebate (gap payment)

### Model coefficients

The coefficient values for the clinical attributes found that overall, individual drugs within both the opioid and NSAID attributes were not statistically significant. However, the attributes of pain reduction (coefficient 0.12, 95%CI 0.02 to 0.22) and adverse events (coefficient − 0.14, 95%CI − 0.20 to − 0.08) were significant, suggesting they individually may influence prescribing choices. The positive coefficient value for pain reduction indicates that for each additional unit reduction in pain intensity, the likelihood to prescribe increases. Whereas the negative coefficient value for adverse events indicates that for each unit increase in adverse events the likelihood to prescribe decreases. Since the patient attributes of leg pain and comorbidities were held fixed when generating the coefficients, the influence of these attributes were not considered in this analysis but were investigated in the analysis of the marginal effects (below). The coefficients are displayed in Table 4.

**Table 4:** Coefficients examining relative importance of each attribute on drug choice

Alternatives and attributes*	Coefficient (95% CI)	P-value	Odds ratio (95% CI)
Opioid			
*Oxycodone*	− 0.155 (− 0.400 to 0.090)	0.215	0.856 (0.670 to 1.094)
*Oxycodone CR*	0.118 (− 0.135 to 0.371)	0.360	1.125 (0.874 to 1.449)
*Tramadol*	− 0.124 (− 0.378 to 0.131)	0.342	0.884 (0.685 to 1.140)
*Codeine/paracetamol*	− 0.003 (− 0.248 to 0.243)	0.982	0.997 (0.780 to 1.275)
NSAID			
*Meloxicam*	0.006 (− 0.202 to 0.214)	0.956	1.006 (0.817 to 1.238)
*Diclofenac*	− 0.002 (− 0.208 to 0.205)	0.988	0.998 (0.812 to 1.228)
*Pain (continuous)*	0.116 (0.017 to 0.215)	0.022**	1.123 (1.017 to 1.240)
*AEs (continuous)*	− 0.142 (− 0.204 to − 0.080)	< 0.001**	0.867 (0.815 to 0.923)
*ASC*	− 1.483 (− 2.092 to − 0.874)	< 0.001**	0.227 (0.123 to 0.417)
Opt out (neither option)			

CR: Controlled release, AE: Adverse Events, ASC: Alternative Specific Constant, NSAID: Non-Steroidal Anti-Inflammatory Drug

*The Alternatives (Opioid, NSAID and Opt out) are in bold and the associated attributes are in italics

**Statistically significant

### Model marginal effects

#### The importance of each attribute in influencing clinical decision making of prescribing (primary outcome)

Overall, the results from the marginal effects found that GPs are more likely to select an NSAID (45.2%, 95% CI 38.7 to 51.7%) compared to an opioid (28.8%, 95% CI 23.0 to 34.7%) to prescribe to a patient with chronic LBP. However, there was no difference (− 0.2%, 95% CI − 2.8 to 2.4%) in the probability of a GP choosing a specific NSAID over another (i.e. either diclofenac or meloxicam). Similarly, if an opioid was chosen, there was no difference in the probability of a GP choosing either oxycodone-controlled release (0.7%, 95% CI − 0.7 to 2.2%), tramadol (1.6%, 95% CI − 1.2 to 4.5%), or codeine and paracetamol (2.7%, 95% CI − 1.5 to 6.9%) over oxycodone immediate release. The probabilities of selecting a specific opioid or NSAID are presented in Table 5.

**Table 5:** Margins examining effects of clinical and patient attributes on drug choice and probabilities of selecting a specific opioid and NSAID

	Alternatives		
	Opioid	NSAID	Opt-out
	Margin (95% CI)	Margin (95% CI)	Margin (95% CI)
*Clinical attributes*			
Drug types			
Any drug type within each category	0.288 (0.230 to 0.347)	0.452 (0.387 to 0.517)	
Oxycodone (immediate release)	0.293 (0.240 to 0.345)		
Oxycodone (controlled release)	0.300 (0.250 to 0.350)		
Tramadol	0.309 (0.259 to 0.359)		
Codeine and paracetamol	0.320 (0.266 to 0.374)		
Meloxicam		0.449 (0.403 to 0.495)	
Diclofenac		0.446 (0.400 to 0.493)	
Pain	0.020 (− 0.009 to 0.050)	− 0.009 (− 0.018 to < 0.001)	− 0.007 (− 0.0162 to 0.001)
Adverse events	− 0.009 (− 0.021 to 0.002)	− 0.001 (− 0.010 to 0.008)	< 0.001 (-0.009 to 0.009)
*Patient attributes*			
Comorbidities			
None	0.303 (0.250 to 0.356)	0.461 (0.412 to 0.509)	0.237 (0.195 to 0.278)
One	0.305 (0.255 to 0.354)	0.452 (0.408 to 0.496)	0.244 (0.206 to 0.281)
Two	0.306 (0.257 to 0.356)	0.443 (0.399 to 0.487)	0.251 (0.213 to 0.288)
Three	0.308 (0.255 to 0.361)	0.434 (0.386 to 0.482)	0.258 (0.215 to 0.301)
Low back pain (LBP)			
LBP without leg pain	0.308 (0.257 to 0.359)	0.443 (0.397 to 0.489)	0.249 (0.209 to 0.290)
LBP with leg pain	0.303 (0.251 to 0.355)	0.452 (0.406 to 0.499)	0.245 (0.205 to 0.284)

NSAID: Non-Steroidal Anti-Inflammatory Drug, NSLBP: Non-specific low back pain, LBP: low back pain, NSAID: Non-Steroidal Anti-Inflammatory Drug

All levels within both patient attributes of LBP with or without leg pain and comorbidities (zero, one, two or three) did not influence the likelihood of a GP selecting an opioid versus an NSAID. The estimates for each level of each patient attribute are presented in Table 5.

#### The influence of pain reduction and adverse events on GPs’ choice of medication (secondary outcome)

When investigating the probabilities of an opioid or NSAID being selected in consideration of pain reduction and adverse events, the results of the marginal effects (Table 5) found these attributes did not significantly influence the specific prescribing choices between and NSAID or opioid analgesic. Therefore, whilst we found that the attributes of pain reduction and adverse events do contribute to a GPs choice to prescribe medicines in general (Table 4), the presence of pain and adverse events did not specifically influence the choice between an NSAID and an opioid for a patient with chronic LBP.

### Sensitivity analyses

A sensitivity analysis was performed by removing participants who selected the “opt-out” choice for every choice set (n = 5 participants) and the analyses repeated. There was no change in the significance of the output when compared with the original logit model and margin analyses. There were also no significant interactions between participant characteristics (presence of leg pain and number of comorbidities) and medication choice after running the post-hoc exploratory analysis of interaction effects.

## Discussion

### Statement of key findings

This study found that GPs preferred to prescribe an NSAID over an opioid analgesic for a patient with chronic low back pain regardless of the presence of comorbidities or leg pain. Additionally, the presence of pain and adverse events did not specifically influence the choice between an NSAID and an opioid for a patient with chronic LBP. There was no preference in the type of NSAIDs or opioids selected.

### Interpretation

Clinical decision-making is dynamic and multi-factorial [49, 50]. Similar to our main finding, previous research in primary care suggests that patient factors may influence GPs’ decisions less than clinical competence or their knowledge of best practice medicine [24, 50, 58–61]. Our results that GPs prefer an NSAID over an opioid is consistent with recent shifts in prescribing data which previously showed increased prescribing of opioid analgesics up until 2017 [18–20, 62, 63]. However, recent evidence [64] is starting see some reduction in opioid prescribing rates [64–66]. The findings from our study and others may suggest greater GP adherence to clinical practice guideline recommendations [67, 69], which now discourage the use of opioid analgesics, and/or that GPs are more aware of the emerging evidence that opioids are no more effective than NSAIDs for LBP [70]. We observed that GP participants in our study were younger (average age was 34 years) and had less experience (average number of years in practice was 6.5 years) than representative samples of general practitioners in Australia [18]. These factors may have influenced our findings as evidence suggests [72] that younger GPs are more likely to base their treatment choices on guideline recommendations than clinical experience.

We also investigated the effect of pain reduction and the likelihood of adverse events, and surprisingly these factors were found to not influence a GP’s prescribing choice. However, previous DCEs [33, 34, 73, 74] found that patients with low back pain generally prefer treatments which provide greater pain reduction, but also consider potential adverse events. Although the effect of patient preferences was not a factor investigated in this study, our findings suggest there are discordances between patient and GP choices [75], which should be explored in future studies.

### Strengths and weaknesses

To our knowledge, no previous DCEs have investigated GP’s choices on analgesic medications for patients with chronic LBP; therefore, this study addressed this gap. We used a D-efficient design and included an ‘opt out’ option, an important design consideration as forcing participants to choose an alternative can otherwise raise concerns over external validity [52]. Our sensitivity analysis confirmed that 2.4% of participants chose to ‘opt-out’ for each question, however these responses did not influence our results. Due to the nature of the DCE design we were limited by the number of attributes and levels we could include and therefore acknowledge important factors we did not investigate including; if patients have a history of opioid use disorder or the clinician’s level of training and/or confidence in prescribing opioid analgesics. However, this feedback was considered in the design phase and our choice set questions were based on weighing up the provision of sufficient information with reducing participant burden in completing the experiment, and sample size requirements.

### Further research

Our DCE only investigated GPs’ stated choices, not their revealed preferences (the difference between what they say they would do and what they actually do). Previous qualitative studies [28–31, 76, 77] have reported GPs’ decisions in real-life situations are often driven by their sense of obligation to help their patients. Therefore, the pressure to satisfy patients’ expectations for pain relief may be an influencing factor on prescribing preferences and should be investigated further. Additionally, future DCEs employing revealed preference techniques would be useful to possibly uncover influences behind GPs choices by observing their actual behaviour, not just their hypothetical preferences.

## Conclusion

GPs prefer to prescribe an NSAID over an opioid for a patient presenting with chronic low back pain regardless of patient factors or comorbidities. However, there was no difference in the type of NSAID or opioid being selected over another. Additionally, pain reduction and adverse events also did not influence a GPs’ choice of medication between either an NSAID or opioid for a patient with chronic LBP.

### Supplementary Information

Below is the link to the electronic supplementary material.

Supplementary file1 (DOCX 17 kb)
