# Supplementary material for: Understanding general practitioners’ prescribing choices to patients with chronic low back pain: a discrete choice experiment
Source: Int J Clin Pharm. 2023 Oct 26;46(1):111–21. doi: 10.1007/s11096-023-01649-y (PMC10831024; doi:10.1007/s11096-023-01649-y)
Supplement: Supplementary file 1 — Supplementary file1 (DOCX 17 kb) [file 11096_2023_1649_MOESM1_ESM.docx]

**Appendix 1: Pilot study results**

***Table A1.1: Pilot study demographic characteristics***

| **Participant characteristics** | **Number of general practitioners (n = 21)** |
| --- | --- |
| **Age category**  < 35 years  35 – 44 years  45 – 54 years  55+ years  Not reported | 12 (57.1%)  6 (28.6%)  2 (9.5%)  0 (0%)  1 (5.8%) |
| **Gender**  Male  Female | 12 (57.1%)  9 (42.9%) |
| **Country of graduation**  Australia  Other | 19 (90.5%)  2 (9.5%) |
| **Years spent in general practice**  < 2 years  2 – 5 years  6 – 10 years  11 – 19 years  20+ years | 0 (0%)  3 (14.3%)  10 (47.6%)  8 (38%)  0 (0%) |
| **Number of general practitioners in the practice**  < 5  > 5 | 9 (42.9%)  12 (57.1%) |
| **Location of practice^*^**  Major city  Inner regional  Outer regional  Remote  Unknown | 20 (95.2%)  0 (0%)  0 (0%)  0 (0%)  1 (5.8%) |
| **Special interest in managing musculoskeletal conditions**  Yes  No | 11 (53.4%)  10 (47.6%) |
| **Workload capacity**  Part-time  Full-time  Retired | 7 (33.33%)  13 (61.9%)  1 (5.8%) |
| **Payment for consultations**  Bulk billed  Gap payment  Both | 13 (61.9%)  4 (19%)  4 (19%) |

*The location of practice was based on the Australian Statistical Geography Standard from The Australian Bureau of Statistics. Volume 5-Remoteness Structure, July 2016.

***Table A1.2: Pilot study process evaluation questions and answers***

| **Question** | **Response (n = 21)** |
| --- | --- |
| Was the information about the study clear? | **Yes:** 20 (95.2%)  **No:** 1 (4.8%) |
| Did the instructions outline clearly, what I needed to do? | **Yes:** 18 (85.7%)  **No:** 3 (14.3%) |
| Was the number of choices set questions to answer manageable? | **Yes:** 19 (90.5%)  **No:** 2 (9.5%) |
| Was it difficult to choose between the options for each of the choice set questions? | **Yes:** 4 (19.1%)  **No:** 17 (80.9%) |
| Is there any other feedback you would like to provide? | **Yes:** 6 (28.6%)*  **No:** 15 (71.4%) |

* **Additional feedback**: Two respondents said that more information about the clinical case is required. Three respondents said it was a very good survey and one respondent said overall the survey was very easy to understand.

**Appendix 2: Process evaluation results from the main DCE**

***Table A2.1: Main study process evaluation questions and answers***

| **Question** | **Response (n = 210)** |
| --- | --- |
| Was the information about the study clear? | **Yes:** 201 (95.7%)  **No:** 9 (4.3%) |
| Did the instructions outline clearly, what I needed to do? | **Yes:** 202 (96.2%)  **No:** 8 (3.8%) |
| Was the number of choice set questions to answer manageable? | **Yes:** 201 (95.7%)  **No:** 9 (4.3%) |
| Was it difficult to choose between the options for each of the choice set questions? | **Yes:** 78 (36.7%)*  **No:** 132 (63.3%) |
| Is there any other feedback you would like to provide? | **Yes:** 7 (3.3%)**  **No:** 203 (96.7%) |

***Reasons for difficulty choosing between options:** Both options would be suitable (n = 3), neither option would be suitable (n = 2), insufficient information was provided on the patient’s medical history (n = 6), the options were not explained clearly (n = 1), clinical uncertainty and difficult to ascertain the benefits and risks (n = 6), not much difference in the outcomes between options (n = 5), not enough options (n = 3), other (n = 9).

** **Additional feedback**: More information is required (n = 4), the questions were too repetitive (n = 2), other (n = 1).
